# Supplementary material for: Genomic Sub-Classification of Ovarian Clear Cell Carcinoma Revealed by Distinct Mutational Signatures
Source: Cancers (Basel). 2021 Oct 19;13(20):5242. doi: 10.3390/cancers13205242 (PMC8533704; doi:10.3390/cancers13205242)
Supplement: Supplementary file 1 [file cancers-13-05242-s001.zip › cancers-1368973-supplementary.pdf]

Supplementary Materials

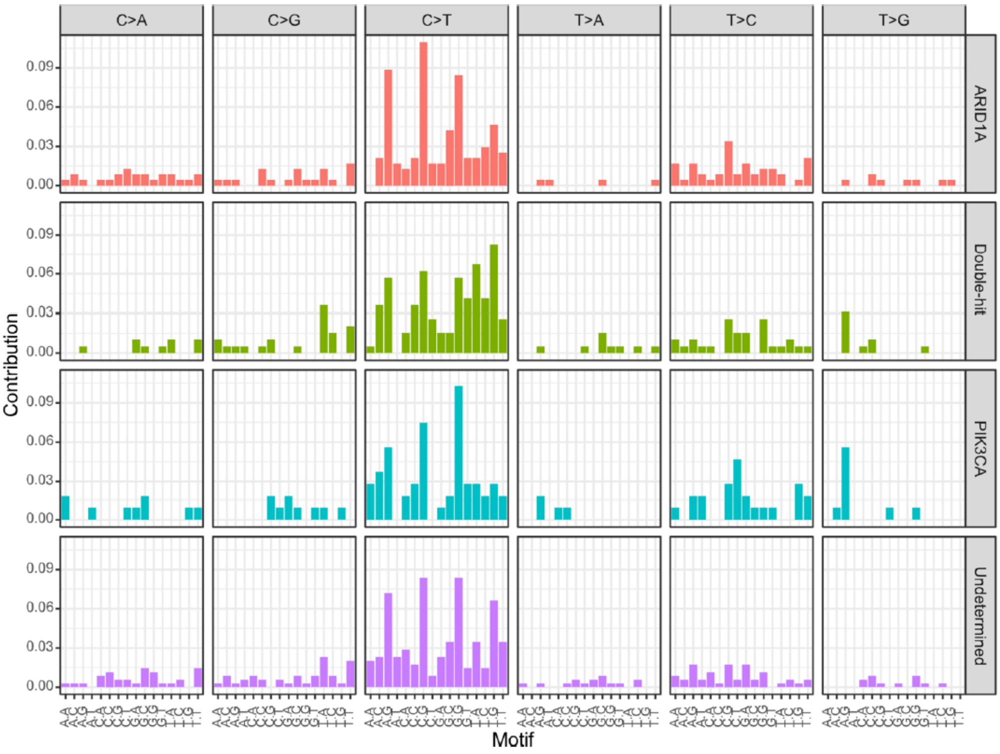

Figure S1. Mutational signatures among the four molecular subgroups.

Table S1. Significant interaction events (co-occurrence or mutually exclusive) among genes.

Gene A	Gene B	P-value	Odds ratio	Type of event	Event ratio
ADAMTS20	*PDE4DIP*	0.0025	20.46	Co_Occurence	4/6
SYNE1	*PIK3CA*	0.0034	0.00	Mutually_Exclusive	0/33
KMT2C	*MYH9*	0.0044	16.34	Co_Occurence	4/7
ARID1A	*PKHD1*	0.0052	0.00	Mutually_Exclusive	0/34
ATM	*KMT2C*	0.0094	10.88	Co_Occurence	4/8
TAF1L	*KMT2C*	0.0094	10.88	Co_Occurence	4/8
CYP2D6	*PBRM1*	0.0112	15.84	Co_Occurence	3/6
TRRAP	*RECQL4*	0.0126	14.14	Co_Occurence	3/6
EP400	*SYNE1*	0.0149	8.96	Co_Occurence	4/9
MYH9	*TAF1L*	0.0212	10.60	Co_Occurence	3/7
KMT2D	*PIK3CA*	0.0244	6.54	Co_Occurence	7/18
TRRAP	*PDE4DIP*	0.0323	8.42	Co_Occurence	3/8
PPP2R1A	*PKHD1*	0.0323	8.42	Co_Occurence	3/8
ATM	*PIK3CA*	0.0338	0.00	Mutually_Exclusive	0/30
PIK3CG	*CSMD3*	0.0463	6.93	Co_Occurence	3/9
PRKDC	*RNF213*	0.0463	6.93	Co_Occurence	3/9

Table S2. Adjusted p-values from multiple comparisons between nucleotide changes among the molecular subgroups.

		ARID1A	Double-hit	PIK3CA	Undetermined
		adjusted p-value			
CA changes	CA/CG	0.93	1	0.72	0.84
	CA/CT	**0.002**	**0.012**	**0.01**	**3.00E-05**
	CA/TA	0.93	1	0.72	0.84
	CA/TC	0.47	1	0.72	0.14
	CA/TG	0.34	1	0.49	0.84
CG changes	CG/CA	1	1	1	0.56
	CG/CT	**0.0022**	**0.0076**	**0.016**	**0.00019**
	CG/TA	1	1	1	0.17
	CG/TC	0.46	1	0.78	0.56
	CG/TG	0.46	0.95	1	0.26
CT changes	CT/CA	**0.002**	**0.003**	**0.0083**	**3.00E-05**
	CT/CG	**0.002**	**0.003**	**0.0096**	**0.00012**
	CT/TA	**0.023**	**0.002**	**0.01**	**4.20E-05**
	CT/TC	**0.018**	**0.00078**	**0.01**	**0.00017**
	CT/TG	**0.0024**	**0.00078**	**0.0016**	**0.00017**
TA changes	TA/CA	1	1	0.72	0.93
	TA/CG	1	1	1	0.13
	TA/CT	0.11	**0.0034**	**0.026**	**5.20E-05**
	TA/TC	0.66	0.8	0.3	**0.0033**
	TA/TG	1	1	1	0.93
TC changes	TC/CA	0.38	0.7	0.39	0.068
	TC/CG	0.38	0.82	0.39	0.31
	TC/CT	**0.045**	**0.00098**	**0.041**	**0.00047**
	TC/TA	0.38	0.6	0.23	**0.0033**
	TC/TG	0.069	0.17	0.081	**0.028**
TG changes	TG/CA	0.26	0.79	0.37	0.64
	TG/CG	0.26	0.71	1	0.26
	TG/CT	**0.004**	**0.00078**	**0.0016**	**0.00042**
	TG/TA	0.62	0.79	1	0.74
	TG/TC	0.069	0.17	0.081	**0.037**
